# A Novel Aptamer-Imprinted Polymer-Based Electrochemical Biosensor for the Detection of Lead in Aquatic Products

**DOI:** 10.3390/molecules28010196

**Published:** 2022-12-26

**Authors:** Nianxin Zhu, Xinna Liu, Kaimin Peng, Hui Cao, Min Yuan, Tai Ye, Xiuxiu Wu, Fengqin Yin, Jinsong Yu, Liling Hao, Fei Xu

**Affiliations:** Shanghai Engineering Research Center of Food Rapid Detection, School of Health Science and Engineering, University of Shanghai for Science and Technology, Shanghai 200093, China

**Keywords:** aptamer-imprinted polymer, chitosan–graphene oxide composite, lead detection

## Abstract

Lead contamination in aquatic products is one of the main hazard factors. The aptasensor is a promising detection method for lead ion (Pb(II)) because of its selectivity, but it is easily affected by pH. The combination of ion-imprinted polymers(IIP) with aptamers may improve their stability in different pH conditions. This paper developed a novel electrochemical biosensor for Pb(II) detection by using aptamer-imprinted polymer as a recognition element. The glassy carbon electrode was modified with gold nanoparticles and aptamers. After the aptamer was induced by Pb(II) to form a G-quadruplex conformation, a chitosan-graphene oxide was electrodeposited and cross-linked with glutaraldehyde to form an imprint layer, improving the stability of the biosensor. Under the optimal experimental conditions, the current signal change (∆I) showed a linear correlation of the content of Pb(II) in the range of 0.1–2.0 μg/mL with a detection limit of 0.0796 μg/mL (S/N = 3). The biosensor also exhibited high selectivity for the determination of Pb(II) in the presence of other interfering metal ion. At the same time, the stability of the imprinted layer made the sensor applicable to the detection environment with a pH of 6.4–8.0. Moreover, the sensor was successfully applied to the detection of Pb(II) in mantis shrimp.

## 1. Introduction

Aquatic products are an important part of people’s daily diet, rich in protein, unsaturated fatty acids, and a variety of trace elements. However, researchers have found that lead (Pb(II)) can be absorbed by and enriched in aquatic product, and eventually accumulated in the human body through transmission in the food chain [1,2], posing a risk to human health. Lead can cause mental retardation [3,4,5], and damage to the nervous system [6,7], bone hematopoietic function [8,9] as well as the reproductive system [10,11]. Thus, the maximum residue limit (MRL) of lead was set to be 0.5 mg/kg in meat, fish, and shrimp by the People’s Republic of China, and 0.3 mg/kg in fish and cephalopods by the European Commission (EU) Regulation No. 1317/2021. However, excessive lead in aquatic products still occurs due to the heavy metal pollution of rivers [12]. Therefore, it is urgent that we detect lead in aquatic products.

At present, the traditional lead ion detection methods are mainly atomic emission spectrometry (AES) [13], inductively coupled plasma mass spectrometry(ICP-MS) [14], and atomic absorption spectrometry (GF-AAS) [15]. Although these detection methods have high accuracy, the instruments are large and require professional operators. Electrochemical analysis has the advantages of a small equipment and easy operation as well as high sensitivity [16,17,18]. Thus, electrochemical biosensors have been used in the detection of metal ions with the recognition elements of antibodies [19,20], aptamers [21,22,23,24,25], and molecular imprinting [26,27,28]. An aptamer is a short oligonucleotide sequence, which has a high affinity and specificity for specific targets, and has the advantages of convenient production and modification in vitro. Nevertheless, the binding performance of aptamers is easily affected by the pH of the buffer, which brings a lot of inconvenience to its use in the detection in food samples. Ion imprinting polymers(IIP) are chemically synthesized through the polymerization of appropriate functional monomer and cross-linking agent to form an imprinted cavity with a complementary 3D shape, combining with the template ions. At the same time, it has the advantage of high stability. On this basis, the combination of an aptamer with IIP may maintain not only the high specificity of the aptamer, but also the stability of the IIP, making it more suitable for real sample detection. Li et al. [29] used L-Alanine as the functional monomer and N-hydroxysuccinimide as the cross-linking agent to prepare an aptamer-imprinted polymer and applied the novel recognition element to detect Cd(II). However, the imprinting process was induced by UV irradiation, which brought inconvenience to the preparation of the aptamer-imprinted polymer.

Electrodeposition is a common electrochemical method [30,31,32]. Herein, in this paper, we used a chitosan–graphene oxide (CS–GO) to fix the spatial conformation of a Pb(II)-aptamer complex on the electrode surface by simple electrodeposition. The working electrode was first modified by an AuNPs and Pb(II)-aptamer compound, and then, the modified electrode was immersed into the CS–GO complex solution followed by the application of the desired voltage for a fixed amount of time. The amine groups of the CS became a cationic polyelectrolyte whose positively charged molecule is attracted toward the negatively charged cathode on the electrode surface. The GO was added to improve the electron transfer efficiency [33] and glutaraldehyde (CHO) acted as a cross-linking agent to form a three-dimensional structure. After removing the Pb(II) with an ethylene diaminetetraacetic acid disodium salt (EDTA), the aptamer-imprinted polymer that specifically binds to Pb(II) was successfully prepared. The results showed that the developed aptamer-imprinted polymer-based electrochemical biosensor possessed good stability and strong specificity in the detection of Pb(II).

## 2. Materials and Methods

### 2.1. Materials and Apparatus

Pb(II) aptamer (5′-SH-GGGTGGGTGGGTGGGT-3′) was obtained from Sangon Biotechnology Co., Ltd. (Shanghai, China). Sodium tetrachloroaurate(III) (HAuCl_4_) and GO was obtained from Sigma-Aldrich (Shanghai, China). Tris (2-carboxyethyl) phosphine (TCEP) and mercaptoethanol (MCH) was purchased from Aladdin Industrial Co. Ltd. (Shanghai, China). Pb (NO_3_)_2_ (Pb(II)), NaCl (Na(I)), KCl (K(I)), CaCl_2_ (Ca(II)), MgCl_2_ (Mg(II)), CdCl_2_ (Cd(II)), CHO (25% in H_2_O), EDTA, acetic acid, and potassium ferricyanide (K_3_ Fe (CN)_6_) were purchased from Sinopharm Reagent Co. Ltd. (Shanghai, China). Ultrapure water with a resistivity of 18.2 MΩ×cm was used in the whole experiment. Phosphate buffer (PBS) solution was acted as a supporting electrolyte (10 mM, contain 0.1 M NaCl, pH 7.4). Mantis shrimp samples were purchased from local seafood market (Shanghai, China).

Electrochemical experiments, such as cyclic voltammetry (CV), electrochemical impedance spectroscopy (EIS), and differential pulse voltammetry (DPV), were performed on a CHI 660 C workstation with a conventional three-electrode system. The working electrode was a glassy carbon electrode (GCE, Ф = 3 mm) or the prepared modified electrode; the auxiliary electrode was platinum wire electrode, and the reference electrode was saturated calomel electrode.

### 2.2. The Preparation of AuNPs-Modified GCE

The GCE was firstly pretreated prior to its modification. The GCE was polished to a mirror-like surface with 0.3 and 0.05 μm alumina for 3 min. Then, it was rinsed with ethanol and water, alternatively, three times each, and was dried by using a nitrogen stream. Then, the GCE was immersed in a 30 mM HAuCl_4_ solution and a constant potential value of −0.8 V was applied for 5 min. The electrode was called AuNPs/GCE.

### 2.3. The Preparation of Aptamer-Imprinted Polymer of Pb(II)

The aptamer-imprinted polymer of Pb(II) was constructed in three steps. Firstly, Pb(II)-aptamer compound was fixed to the surface of AuNPs. The Pb(II) aptamer was activated at 95 °C for 10 min and then mixed with 100 μM TCEP for 1 h to open the disulfide bond, and the aptamer was immobilized on the surface of AuNPs/GCE electrode by incubating the electrode in 30 μL of 10 μM atpamer solution for 12 h at 25 °C. The aptamer/AuNPs/GCE was immersed in 30 μL of 1 mM MCH for 20 min to eliminate the nonspecific adsorption of the aptamer. Then, the aptmer/MCH/AuNPs/GCE was immersed in 20 μL of 15 μM Pb (NO_3_)_2_ solution and reacted for 0.5 h to induce the spatial conformation of aptamer.

Secondly, CS–GO complex was fixed around Pb(II)-aptamer compound to form an imprinted layer by electrodeposition. 12 mg CS and 0.6 mg GO were added to the 1% acetic acid solution, with stirring for 2 h to form the complex of CS–GO. The CS–GO complex was deposited on the surface of the Pb(II)/aptmer/MCH/AuNPs/GCE by the one-step deposition method with the deposition voltage of −1.2 V and the deposition time of 20 s. Then, the electrodeposition film of CS–GO/Pb(II)/aptmer/MCH/AuNPs/GCE was cross-linked with 0.25% CHO solution for 0.5 h.

Finally, Pb(II) was extracted from imprinted texture by EDTA. The treated electrode was eluted in 0.5 M EDTA solution for 2 h. After every step, the obtained electrode was washed with water several times to remove residue and was dried by using a nitrogen stream. The Pb(II) aptamer-imprinted polymer (aptamer-IIP-Pb(II)) modified electrode was obtained. For comparison, the non-aptamer-imprinted polymer-modified electrode designated as aptamer-NIP-Pb(II) was prepared by the same method but in the absence of Pb(II).

### 2.4. Electrochemical Measurements

CV and EIS were carried out in 5 mM [Fe (CN)_6_]^3–^/^4–^ and 0.1 M KCl solution. The voltage range of CV was 0.0 V to + 0.4 V, and the scanning speed was 50 mV/s. The frequency range of EIS was 0.1 to 10 kHz, and the voltage was 0.25 V. The electrochemical detection was carried out by DPV with a scanning range of 0 V to −0.6 V and an amplitude of 50 mV.

### 2.5. The Pretreatment of Food Samples

For the real sample analysis, mantis shrimp samples are used to evaluate the appropriateness of the developed sensor for detecting lead. Three Pb(II) solutions with different concentrations (1, 10, and 20 μg/mL) were added to mantis shrimp samples and mixed for 30 min. 6 mL HNO_3_ (68%, ω) and 2 mL H_2_O_2_ (30%, ω) were added to the samples and digested by using a microwave digestion system (MARS6, CEM, Matthews, NC, USA). In the first step of the digestion, the temperature increased to 120 °C over 10 min. In the second step, the temperature increased from 120 to 190 °C over 10 min, and then held constant for further 30 min. After digestion, the samples were cooled to about 25 °C. The above mixture was then adjusted to pH of 7.0 and diluted by PBS with the final concentrations of Pb(II) of 0.1, 1, and 2 μg/mL.

## 3. Results and Discussion

### 3.1. The Principle of the Developed Aptamer-Imprinted Polymer-Based Biosensor for the Detection of Pb(II)

**Scheme 1:**
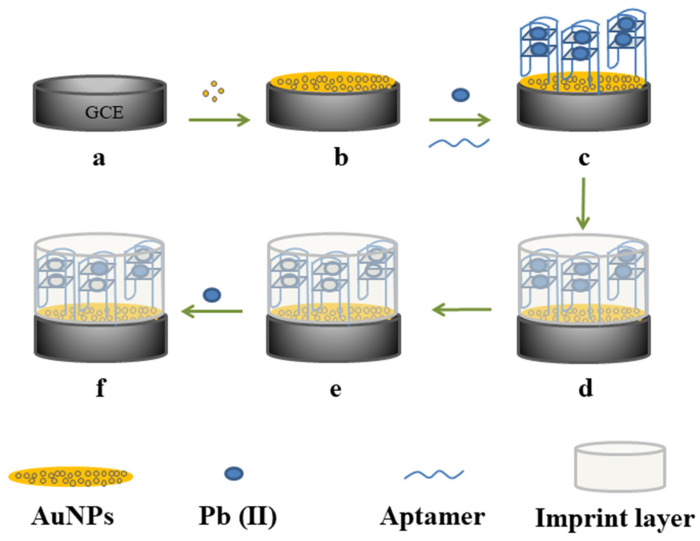
Schematic diagram of aptamer-IIP-Pb(II) preparation for detection of Pb(II). (**a**) GCE; (**b**) electrodeposition of AuNPs on bare GCE; (**c**) on AuNPs/GCE surface, attachment of Pb(II)-aptamer complex; (**d**) on Pb(II)/Aptmer/MCH/AuNPs/GCE surface, formation of imprinted layer; (**e**) removal of Pb(II) with EDTA; (**f**) adding Pb(II) as a target.

Scheme 1 provided a schematic diagram of the principle of the developed aptamer-imprinted polymer-based biosensor for the detection of Pb(II). Firstly, AuNPs were uniformly coated on the electrode surface to increase the surface area of the electrode. Secondly, the aptamer was fixed on the surface of the AuNPs through an Au–S bond. After the combination of the aptamer with Pb(II), the special conformation of aptamer-Pb(II) was then fixed by the electrodeposition of the CS–GO complex and cross-linked by CHO. Finally, the Pb(II) in the imprinted polymer was eluted by EDTA, leaving specific cavities on the surface of the working electrode to obtain the aptamer-imprinted polymer for Pb(II). In the presence of Pb(II), it would bind to the specific cavities of the aptamer-imprinted polymer. The Pb(II) enriched on the electrode was reduced when the working electrode voltage scanned from positive to negative, causing changes in the current signal (∆I) that measured by the DPV method. The higher the concentration of Pb(II) in the solution, the more the Pb(II) could be enriched, and the higher the value of ∆I. Therefore, the concentration of Pb(II) could be measured by detecting the ∆I value.

### 3.2. The Morphology Characterization of the Prepared Electrodes

The morphology of AuNPs/GCE and aptamer-IIP-Pb(II)/AuNPs/GCE were characterized by SEM. As shown in Figure S1A, the AuNPs have been uniformly wrapped on the electrode surface. The CS–GO was electrodeposited onto the surface of the aptamer-Pb(II) complex and reacted with CHO to form an imprinted layer. The SEM image (Figure S1B) showed that the gaps between the AuNPs were filled with the imprinted polymer, and the electrode surface was more compact, indicating the successful preparation of an aptamer-IIP-Pb(II)-modified electrode [34].

### 3.3. The Electrochemical Characterization of the Fabricated Biosensor

**Figure 1:**
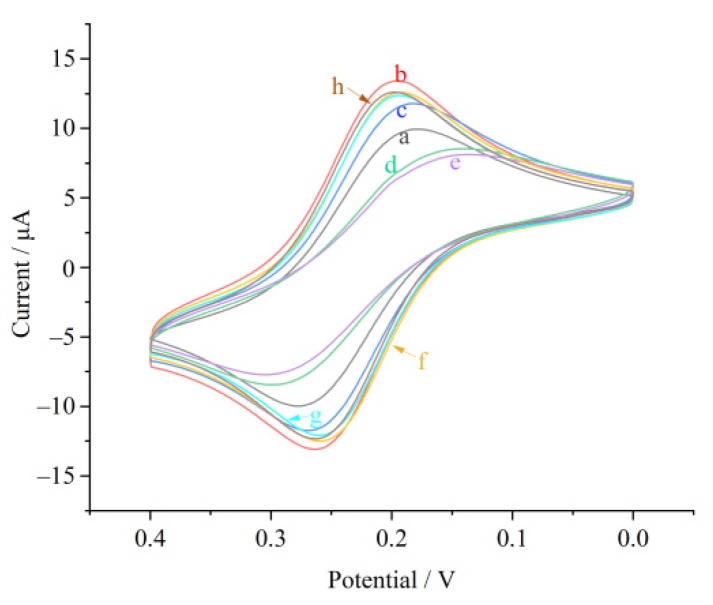
Typical CV curves of (a) bare GCE; (b) AuNPs/GCE; (c) aptamer/AuNPs/GCE; (d) Aptmer/MCH/AuNPs/GCE; (e) Pb(II)/Aptmer/MCH/AuNPs/GCE; (f) CS–GO/Pb(II)/Aptmer/MCH/AuNPs/GCE; (g) CHO/CS–GO/Pb(II)/Aptmer/MCH/AuNPs/GCE; (h) aptamer-IIP-Pb(II) in 5 mM [Fe (CN)_6_]^3−^/^4−^ and 0.1 M KCl.

The layer-by-layer changes on the surface properties of the electrode could be evaluated by CV and EIS in 5 mM [Fe (CN)_6_]^3–^/^4–^ and 0.1 M KCl solutions. Figure 1 and Figure S2 showed the changes of the electrode electrochemical properties during the modification process.

In Figure 1, Curve a showed the conductivity of GCE. A pair of clear reversible redox peaks could be observed, indicating the reversible electrochemical process. After the AuNPs were fixed on the GCE surface, the peak current (Curve b) increased significantly. This phenomenon could be attributed to the excellent electron transfer performance of AuNPs. After the aptamer was immobilized on the surface of the AuNPs by an Au–S bond, the peak current decreased significantly (Curve c). This might be due to the repulsion between the phosphate group of the aptamer and the negative charge of the redox probe. After blocking the electrode with MCH, the empty sites on the AuNPs surface were occupied, which further hindered the electron transmission and led to the decrease of the peak current (Curve d). The Pb(II) induced the DNA aptamers to form G-quadruplexes, which further increased the steric hindrance and led to poor electron transfer performance. Therefore, Curve e showed that the peak current further decreased. When the electrodeposition of the CS–GO was carried out on the surface of the G-quadruplex-Pb(II) compound, the peak current increased significantly due to the excellent electronic conductivity of GO (Curve f). The addition of the cross-linking agent CHO formed IIP cavities with the G-quadruplex-Pb(II) compound as a template, so a successful decrease in the peak current was observed (Curve g). The formation of an aptamer-IIP complex and the steric/conformational restriction between the modified surface and the redox probe hindered the electron transfer. After cleaning the template with EDTA, a slight recovery of the peak current was observed (Curve h) because the Pb(II) was eluted and the imprinted cavities made the electron transfer ability stronger. These results indicated that the aptamer-IIP-Pb(II)-modified electrode was successfully prepared.

EIS is another technology to characterize the electron transfer characteristics of different modified electrodes, and the charge transfer resistance R_ct_ is the key parameter of this technology. The Nyquist curves of the modified electrodes at different stages were shown in Figure S2. The EIS data confirmed the above conclusions obtained by CV. Both of them proved that the aptamer-IIP-Pb(II)-modified electrode was successfully prepared.

### 3.4. Optimization of Experimental Conditions

The concentration of the aptamer and the thickness of the ion-imprinted layer may affect the performance of the aptamer-IIP-Pb(II) biosensor. The optimum experimental conditions were obtained by investigating the electrodeposition time of the CS–GO and the concentration of the aptamer.

**Figure 2:**
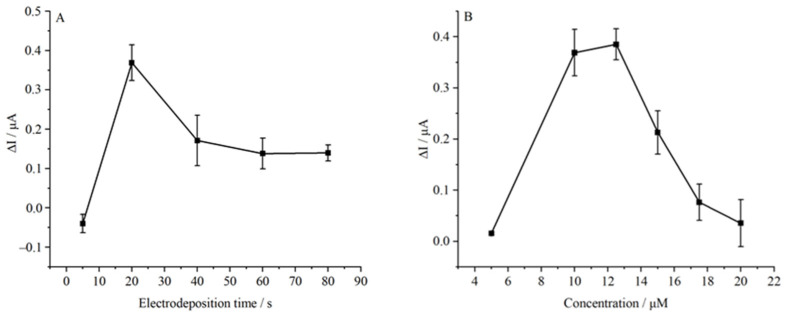
Effects of electrodeposition time of CS–GO (**A**) and the concentration of aptamer (**B**) on the DPV response of IIP-aptamer-Pb(II) toward 1 μg/mL Pb(II) in 0.1 M phosphate buffer.

The CS–GO was modified to the surface of Pb(II)/Aptmer/MCH/AuNPs/GCE by the amperometric i-t curve (i-t) method with different electrodeposition times (5, 20, 40, 60, and 80 s) with an aptamer concentration of 10 μM. The current difference (∆I = I − I_0_, where I represents the sample current value, and I_0_ represents the blank current value) was acquired by the DPV method. The results were displayed in Figure 2A. The best electrodeposition time was obtained in 20 s. With the increase of electrodeposition time, the more CS–GO was deposited on the electrode. Before 20 s, there was less CS–GO, so the imprinted layer formed could not fix the G-quadruplex well. In the process of elution and detection, unfixed aptamers were easily affected by the solution environment (such as organic reagents and solution pH), and their ability to capture Pb(II) was limited. After more than 20 s, more CS–GO was electrodeposited on the modified electrode, which then reacted with CHO to form an imprinted layer. The overly thick imprinted layer made it difficult for the Pb(II) to elute and adsorb again, resulting in the peak current signal weakening below 0.2 μA.

The aptamer was modified to the surface of AuNPs/GCE by immersing the electrode in an aptamer solution with different concentrations (5, 10, 12.5, 15, 17.5, and 20 μM) and leaving it standing for 12 h, with the electrodeposition time of CS–GO set to 20 s. The results were displayed in Figure 2B. With the increase of the concentration of the aptamer, the signal peak current first increased and then decreased, reaching a maximum of 12.5 μM. When the concentration of the aptamer was low, the aptamer on the electrode surface did not reach saturation, so its ability to capture Pb(II) increased with the increase of the concentration of the aptamer. When the concentration of the aptamer exceeded 12.5 μM, the aptamer stacking on the electrode surface was excessive. The Pb(II) induced the aptamers to form a G-quadruplex, which increased the steric hindrance. Crowded aptamers would hinder the formation of the G-quadruplex, thus reducing the amount of Pb(II) captured by the aptamers and decreasing the peak current signal.

### 3.5. Analytical Performance of the Developed Biosensor

**Figure 3:**
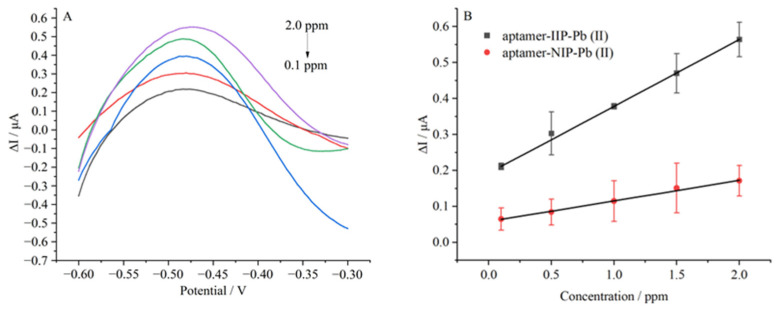
Typical DPV curves (**A**) of IIP-aptamer-Pb(II) in pH 7.4 phosphate buffer with different concentrations of Pb(II), respectively; linear relationship (**B**) between the peak current and the concentration of Pb(II).

The limit of detection (LOD) and linear range were studied under the optimal conditions by using the same series of Pb (NO_3_)_2_ solutions with different concentrations. The aptamer-IIP-Pb(II) was incubated in Pb (NO_3_)_2_ solutions of different concentrations (0.1 to 2.0 μg/mL) for 30 min. The DPV response was shown in Figure 3A. A gradual increase of the current signal of the aptamer-IIP-Pb(II) was observed with increasing concentrations of Pb(II) in the electrolyte solution. The plot of ∆I (∆I = I − I_0_, where I represents the sample current value, and I_0_ represents the blank current value) versus the concentration of Pb(II) presented an excellent linear relationship in the detection range of 0.1 to 2.0 μg/mL (Figure 3B). The regression equation was ∆I (μA) = 0.1927 + 0.1857 C_Pb(II)_ (μg/mL) (R^2^ = 0.9996). The LOD was calculated as 0.0796 μg/mL at a signal-to-noise ratio (S/N) of 3 according to the following equation: LOD = 3δ/K, where LOD was the detection limit at the 95% confidence level, δ was the standard deviation of the blank measurements, and K was the slope of the calibration curve. The performance of the developed biosensor was compared with other reported methods (Table S1). The results showed that the LOD value and linear range dynamic of the developed biosensor were similar to the other methods. It was noteworthy that the aptasensor had excellent selectivity. On this basis, the spatial conformation of the aptamer-Pb(II) was fixed by an imprinting process, which increased the stability of the sensor and could capture and detect Pb(II) in a wider range of pH. Therefore, it might be more convenient for the detection of real samples.

The imprinting factor(IF) was calculated as follows: IF = ∆I_(IIP)_/∆I_(NIP)_, where ∆I_(IIP)_ and ∆I_(NIP)_ were the current response values of the aptamer-IIP-Pb(II) and aptamer-NIP-Pb(II) after a template recombination by the DPV method. The ∆I_(IIP)_ and ∆I_(NIP)_ under different concentrations of Pb(II) were shown in Figure 3B. The IF of this biosensor was 3.25. The results showed that the aptamer-IIP-Pb(II) had a specific recognition ability for Pb(II) compared with aptamer-NIP-Pb(II).

### 3.6. Selectivity of the Developed Biosensor

**Figure 4:**
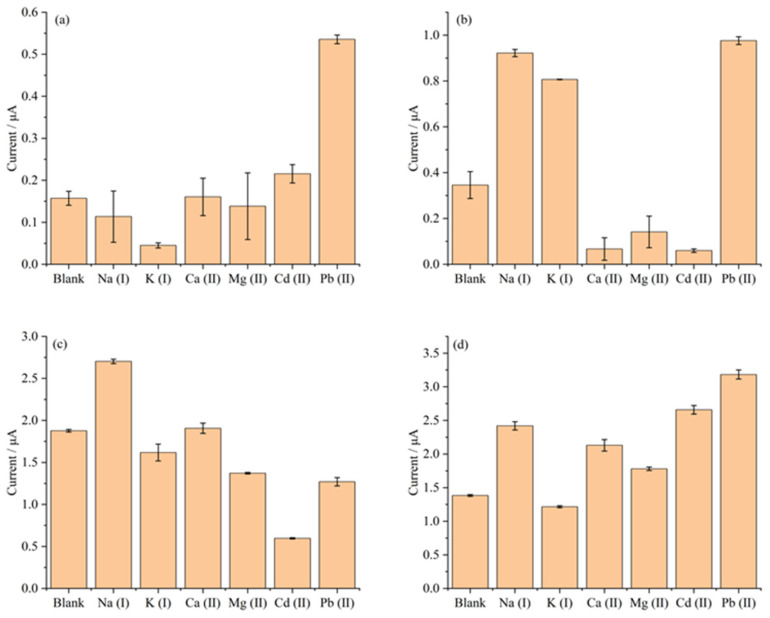
Selectivity of aptamer-IIP-Pb(II) (**a**), aptamer-Pb(II) (**b**), IIP-Pb(II) (**c**), and CS–GO (**d**) sensors for detecting 1 μM Pb(II).

Selectivity is one of the most critical performances of a biosensor which can discriminate between the interfering metal ions with similar positive charge and structures. In this part, the selectivity of the developed aptamer-imprinted polymer biosensor based on aptamer-IIP was tested using other similar or possible co-existent metal ions. As shown in Figure 4a, neglectable DPV responses were observed with the addition of interfering metal ions, indicating that the developed biosensor possesses excellent selectivity towards Pb(II). This was mainly due to the excellent selectivity of the prepared aptamer-IIP polymer. In addition, the selectivity of the aptamer, the IIP-Pb(II) sensor, and the CS–GO sensor were also tested. The aptasensor was prepared by modifying the aptamer on the AuNPs/GCE electrode. The IIP-Pb(II) sensor was prepared by electrodepositing the CS–GO complex and Pb(II) on the AuNPs/GCE electrode, cross-linking with CHO and eluting the Pb(II) with EDTA, while the CS–GO sensor was prepared by the same method but in the absence of Pb(II). It could be seen in Figure 4 that the selectivity performance of the aptamer was worse than the aptamer-IIP polymer, which might be because of the affinity of the G-quadruplex to Na(I) and K(I). Furthermore, the selectivity performances of the IIP (Figure 4c) and CS–GO (Figure 4d) towards Pb(II) were also worse than the aptamer-IIP polymer, because the CS had a large number of active functional groups such as amino, hydroxyl, and N-acetyl groups in its molecular chain segment, which could adsorb and co-ordinate various metal ions. The above selectivity results demonstrated that the aptamer-imprinted polymer strategy was proven to be a promising alternative approach for improving the selectivity of the aptamer.

### 3.7. Stability of the Developed Biosensor

Because the formation and maintenance of the spatial conformation of aptamers require specific pH, the good stability of the aptasenor typically needs specific pH conditions. Thus, in this section, we evaluated the stability of the developed aptamer-imprinted polymer biosensor based on the aptamer-IIP in different pH conditions by comparing the performances of the biosensors based on the aptamer and CS–GO IIP.

**Figure 5:**
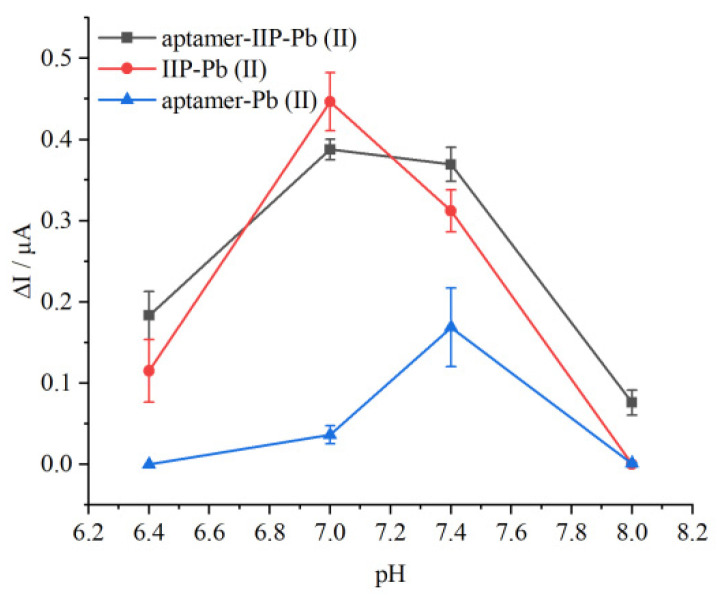
Stability of aptamer-IIP-Pb(II), aptamer-Pb(II), and IIP-Pb(II) sensors for detecting 1 μM Pb(II) in different pH (6.4, 7.0, 7.4m, and 8.0).

As shown in Figure 5, compared with the aptasensor and CS–GO sensor, the aptamer-IIP biosensor presented a trend towards higher ∆I values (∆I = I − I_0_, where I represents the sample current value, and I_0_ represents the blank current value) from pH 6.4 to pH 8.0, indicating wider pH adaptability. The spatial conformation of the aptamer-Pb(II) was susceptible to the pH, which, thus, influences the stability of the aptasensor. Therefore, we used the aptamer-Pb(II) complex as the imprinting template during the imprinting process to fix the spatial conformation of the aptamer. The relatively stable rigid structure of the aptamer-Pb(II)-imprinted polymer may be the main reason for the increase in the stability under different pHs. Thus, the detecting performance of the developed biosensor proved to be less affected by the pH, suggesting that the prepared aptamer-imprinted polymer possessed better stability in different pH conditions and might have a better application prospect in real food samples with a simple pretreatment.

### 3.8. Application in Real Samples

In order to investigate the application of the aptamer-IIP-Pb(II) sensor in real samples, the standard addition and recovery experiments were carried out for the sample prepared in Section 2.5. The consumption of mantis shrimps is huge. However, it was reported that the lead content in mantis shrimps could easily exceed the standard. Therefore, mantis shrimps were used as the real sample in this study. We spiked mantis shrimp samples with Pb (NO_3_)_2_ solutions of different concentrations: 0.1, 1.0, and 2.0 μg/mL. As shown in Table 1, the obtained recoveries of the developed method were between 79.74 to 97.93%, with an RSD between 0.78 to 4.51%, which was consistent with ICP-MS test results. The standard addition and recovery results illustrated that our sensor had a good accuracy and repeatability for the detection of Pb(II) in mantis shrimps.

## 4. Conclusions

In this study, an electrochemical biosensor for the sensitive detection of Pb(II) was developed to improve the stability of the aptasensor, using an aptamer-imprinted polymer as a novel recognition element. In this strategy, a chitosan and graphene oxide complex were used to fix the spatial conformation of the Pb(II) aptamer on the electrode surface by simple electrodeposition, and glutaraldehyde was used as a cross-linker to form the Pb(II) aptamer-imprinted polymer. When the Pb(II) in the imprinted polymer was eluted by EDTA, the specific cavities of the aptamer-imprinted polymer for Pb(II) were obtained. The Pb(II) bound to the specific cavities were reduced when the working electrode voltage scanned from positive to negative, leading to changes in the current signal. The results of this study revealed that, under optimal experimental conditions, our biosensor exhibited high sensitivity with a Pb(II) detection limit of 0.0796 μg/mL. Furthermore, compared with the aptasensor and IIP biosensor, the developed electrochemical biosensor proved to possess better performance of selectivity and stability at different pH conditions, which might be mainly due to the construction of the aptamer-imprinted polymer. Moreover, the biosensor was also successfully used to detect Pb(II) in mantis shrimps, and the results were consistent with ICP-MS, demonstrating that the developed electrochemical biosensor had good accuracy and could be successfully applied to real samples.

## Figures and Tables

**Table 1:** Determination of Pb(II) in three mantis shrimp samples (*n* = 3).

Sample	Spiked Concentration (μg/mL)	Pb(II) Concentration Determined by ICP-MS (μg/mL)	Recovery (%)	Pb(II) Concentration Determined by Sensor (μg/mL)	Recovery (%)	RSD (%)
1	0.0	0.018	/	ND	/	/
2	0.1	0.109	109.00	0.08	79.74	2.95
3	1.0	1.081	108.10	0.96	95.75	0.78
4	2.0	2.288	114.40	1.96	97.93	4.51

## Data Availability

Not applicable.

## Supplementary Materials

The following supporting information can be downloaded at: https://www.mdpi.com/article/10.3390/molecules28010196/s1, Figure S1: SEM images of the surface of AuNPs/GCE (A), and aptamer-IIP-Pb(II)/AuNPs/GCE (B) in 2 μm scale; Figure S2: EIS Nyquist plots of (a) bare GCE; (b) AuNPs/GCE; (c) aptamer/AuNPs/GCE; (d) Aptmer/MCH/AuNPs/GCE; (e) Pb(II)/Aptmer/MCH/AuNPs/GCE; (f) CS-GO/Pb(II)/Aptmer/MCH/AuNPs/GCE; (g) CHO/CS-GO/Pb(II)/Aptmer/MCH/AuNPs/GCE; (h) aptamer-IIP-Pb(II) in 5 mM [Fe (CN)_6_]^3−^/^4−^ and 0.1 M KCl; Table S1: Comparison of analytical response of biological devices in Pb(II) detection with other reported analytical methods. References [35,36,37,38] are cited in the supplementary materials.
