# Supplementary material for: A Novel Aptamer-Imprinted Polymer-Based Electrochemical Biosensor for the Detection of Lead in Aquatic Products
Source: Molecules. 2022 Dec 26;28(1):196. doi: 10.3390/molecules28010196 (PMC9822230; doi:10.3390/molecules28010196)
Supplement: Supplementary file 1 [file molecules-28-00196-s001.zip › molecules-2093826-supplementary.pdf]

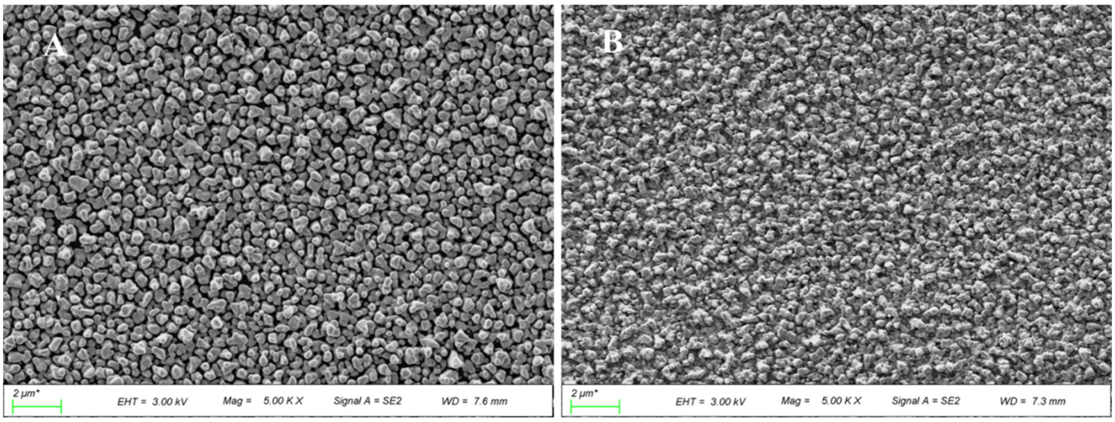

Figure S1 SEM images of the surface of AuNPs/GCE (A), and aptamer-IIP-Pb (II)/AuNPs/GCE (B) in 2 μm scale.

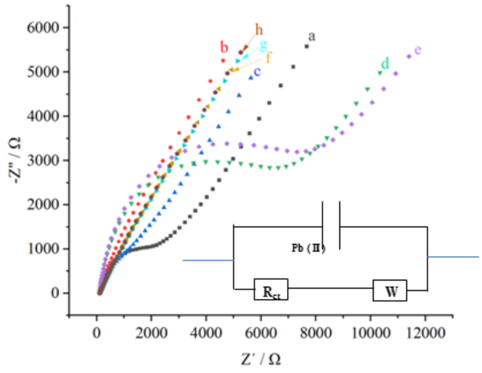

Figure S2 EIS Nyquist plots of (a) bare GCE; (b) AuNPs/GCE; (c) aptamer/AuNPs/GCE; (d) Aptmer/MCH/AuNPs/GCE; (e) Pb (II)/Aptmer/MCH/AuNPs/GCE; (f) CS-GO/Pb (II)/Aptmer/MCH/AuNPs/GCE; (g) CHO/CS-GO/Pb (II)/Aptmer/MCH/AuNPs/GCE; (h) aptamer-IIP-Pb (II) in 5 mM $[Fe (CN)_6]^{3-/4-}$ and 0.1 M KCl.

As shown in Fig. S2, when AuNPs were deposited on the activated GCE electrode (curve a), a very small resistance was observed (curve b) because of their very good conductivity. When the aptamer was fixed to the surface of AuNPs (curve c), the R_{ct} increased significantly, which meant that electron transfer became difficult. When the remaining sites on the surface of AuNPs were occupied by MCH (curve d), R_{ct} increased further. Pb (II) induced the aptamer to form G-quadruplexes (curve e), leading the steric hindrance increased and the electron transfer restricted, so the R_{ct} was further increased. Because of the good conductivity of GO, when CS-GO mixture was electrodeposited on the electrode surface, the resistance was very small (curve f). The aldehyde group of CHO reacted with the amino group of CS to form Schiff base (curve g), which limited the electron transfer on the electrode surface, so the R_{ct} increased. Finally, the template ions

were eluted (curve h), resulting in stronger electron transfer and weaker R_{ct} on the surface of the modified electrode.

Table S1 Comparison of analytical response of biological devices in Pb (II) detection with other reported analytical methods.

Electrode	Linear range (μg/mL)	LOD (μg/mL)	Working solution	Real sample	Ref.
MnFe2O4/GO/GCE	0.066-0.364	0.029	pH 5.0 PBS	river water	[1]
Fe3O4@SiO2@IIP@GCE	0.033-26.497	0.0166	pH 5.6 PBS	tap water/river water/rain water/ fruit juice	[2]
α-MnO2/GCE	0.099-0.397	0.0238	pH 5.0 PBS	/	[3]
SbF/GO/SPCE[a]	0.033-0.497	0.009	0.5 M HCl	sewage/fertilizer waste/sea water	[4]
IIP/aptamer/GCE	0.100-2.000	0.0796	pH 7.0-7.4 PBS	mantis shrimp	this work

[a] The antimony film-graphene oxide modified screen-printed carbon electrode.

References

1. Zhou, S.; Han, X.; Fan, H.; Huang, J.; Liu, Y., Enhanced electrochemical performance for sensing Pb (II) based on graphene oxide incorporated mesoporous $MnFe_2O_4$ nanocomposites. *Journal of Alloys Compounds* **2018,** *747*, 447-454.
2. Dahaghin, Z.; Kilmartin, P. A.; Mousavi, H. Z., Novel ion imprinted polymer electrochemical sensor for the selective detection of lead (II). *Food chemistry* **2020,** *303*, 125374.
3. Zhang, Q.; Peng, D.; Huang, X., Effect of morphology of α-MnO_2 nanocrystals on electrochemical detection of toxic metal ions. *Electrochemistry communications* **2013,** *34*, 270-273.
4. Ruengpirasiri, P.; Punrat, E.; Chailapakul, O.; Chuanuwatanakul, S., Graphene Oxide-Modified Electrode Coated with in-situ Antimony Film for the Simultaneous Determination of Heavy Metals by Sequential Injection-Anodic Stripping Voltammetry. *ELECTROANALYSIS* **2017,** *29* (4), 1022-1030.
